# Lipid Nanoparticles Enable Efficient In Vivo DNA Knock-In via HITI-Mediated Genome Editing

**DOI:** 10.3390/biom14121558

**Published:** 2024-12-06

**Authors:** Jun Hirose, Emi Aizawa, Shogo Yamamoto, Mingyao Xu, Shigenori Iwai, Keiichiro Suzuki

**Affiliations:** 1 Graduate School of Engineering Science, Osaka University, Toyonaka 560-8531, Osaka, Japan; hirose@bio.chem.es.osaka-u.ac.jp (J.H.); u213131f@ecs.osaka-u.ac.jp (S.Y.); iwai.s.es@osaka-u.ac.jp (S.I.); 2 Graduate School of Frontier Bioscience, Osaka University, Suita 565-0871, Osaka, Japan; u717187g@ecs.osaka-u.ac.jp; 3 Institute for Advanced Co-Creation Studies, Osaka University, Toyonaka 560-8531, Osaka, Japan

**Keywords:** genome editing, lipid nanoparticle (LNP), homology-independent targeted integration (HITI), knock-in

## Abstract

In vivo genome editing holds great therapeutic potential for treating monogenic diseases by enabling precise gene correction or addition. However, improving the efficiency of delivery systems remains a key challenge. In this study, we investigated the use of lipid nanoparticles (LNPs) for in vivo knock-in of ectopic DNA. Our in vitro experiments demonstrated that the homology-independent targeted integration (HITI)-mediated genome-editing method achieved significantly higher knock-in efficiency at the *Alb* locus in hepatic cells compared to the traditional homology-directed repair (HDR)-mediated approach. By optimizing LNP composition and administration routes, we successfully achieved HITI-mediated GFP knock-in (2.1–2.7%) in the livers of mice through intravenous delivery of LNP-loaded genome editing components. Notably, repeated intravenous dosing led to a twofold increase in liver GFP knock-in efficiency (4.3–7.0%) compared to a single dose, highlighting the potential for cumulative genome editing effects. These findings provide a solid foundation for the use of LNPs in in vivo knock-in strategies, paving the way for future genome-editing therapies.

## 1. Introduction

CRISPR-Cas9 is one of the most powerful genome-editing tools, enabling precise gene insertion, known as knock-in, to correct pathogenic mutations. Delivering genome-editing components directly to target organs in vivo holds immense promise for proving lasting therapeutic benefits for inherited diseases [1,2,3,4]. Beyond gene correction, CRISPR-Cas9 also allows for the insertion of exogenous genes into safe genomic loci, offering a durable solution for diseases caused by gene loss of function [5,6].

CRISPR-Cas9 induces a double-strand break (DSB) in DNA, triggering two primary DSB repair pathways that can be exploited for both gene knock-ins and knockout: homology-directed repair (HDR) and non-homologous end joining (NHEJ) [7]. HDR, which requires a donor template with homology arms for recombination, has traditionally been the preferred method for knock-ins. However, HDR is restricted to the S-G2 phase of dividing cells, limiting its effectiveness in tissues with low proliferation rates. Additionally, HDR often exhibits low knock-in efficiency [3]. In contrast, NHEJ, the other major DSB repair mechanism, rapidly joins broken DNA ends without the need for homology. This process, which frequently introduces insertions or deletions, is a key tool for gene knockouts. Unlike HDR, NHEJ functions throughout most of the cell cycle, enabling gene knockouts not only in dividing cells but also in non-dividing cells [8].

**Figure A1:**
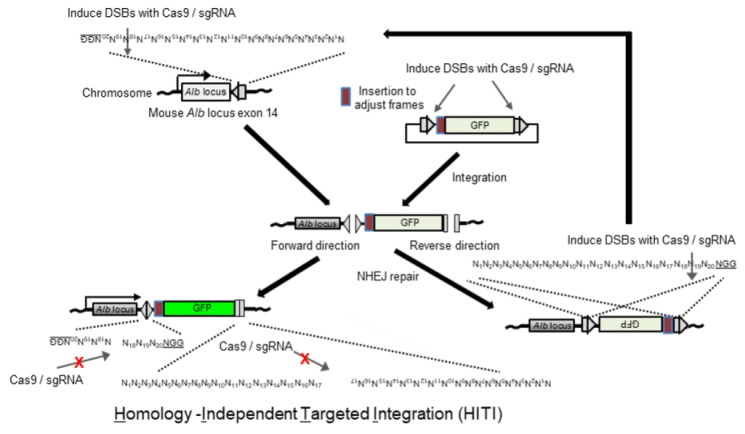
Schematic of HITI-mediated knock-in of GFP coding gene to *Alb* locus.

To leverage the high repair activity and cell cycle independence of NHEJ, we previously developed the homology-independent target integration (HITI) method [9]. This approach capitalizes on NHEJ to offer a versatile and efficient alternative for knock-in applications. A key feature of HITI is the inclusion of Cas9-targeted single guide RNA (sgRNA) sequences in the donor vector as reverse complements to the target site, allowing the system to cleave and correct misoriented insertions until the correct orientation is achieved (Appendix A Figure A1). Notably, HITI enables in vivo knock-in across diverse tissues and organs, including the liver, heart, muscle, and retina [10,11,12,13,14]. NHEJ-mediated knock-in, including HITI, also supports the insertion of large DNA fragments, up to 50 kb [10]. This capacity to target multiple organs and insert large DNA sequences is particularly valuable for therapeutic protein production in vivo, such as inserting genes encoding large proteins like antibodies (~150 kDa) directly into target tissues and organs.

Both HDR- and HITI-mediated knock-ins require the efficient delivery of donor DNA to target cells. Viral vectors, such as adeno-associated viruses (AAV), have predominantly been used to deliver genome-editing components, including Cas9, sgRNA, and donor DNA, for in vivo gene correction or addition [1]. However, AAV systems present several limitations, including immune responses, restricted cargo capacity, and challenges in large-scale manufacturing [15,16]. In contrast, non-viral delivery systems, particularly lipid nanoparticles (LNPs), offer distinct advantages, such as reduced immunogenicity and the ability to enable repeated dosing without loss of efficacy [17,18,19]. LNPs have remarkable success in delivering siRNA and mRNA, with real-world applications like the COVID-19 vaccine and treatment of transthyretin amyloidosis. Thus, LNPs are expected to become scalable, cost-effective, and versatile tools for in vivo genome editing as well [17].

Despite significant progress in LNP-mediated delivery of siRNA and mRNA in vivo, reports on LNP-mediated DNA delivery remain scarce [18,19]. Consequently, most LNP-based genome editing strategies have focused on gene knockout, typically by delivering Cas9 and sgRNA in mRNA or ribonucleoprotein (RNP) forms [17]. Achieving gene knock-in, however, requires the delivery of donor DNA, a largely untapped potential for LNPs [17]. While recent studies have explored LNPs loaded with short single-strand DNA (ssDNA) for in vivo knock-in, they have only succeeded in inserting short fragments (~20 bp) via HDR [20,21]. Hybrid systems that combine Cas9-mRNA- and sgRNA-loaded LNPs with AAV-delivered donor DNA are still required for large DNA knock-ins [22].

**Figure 1:**
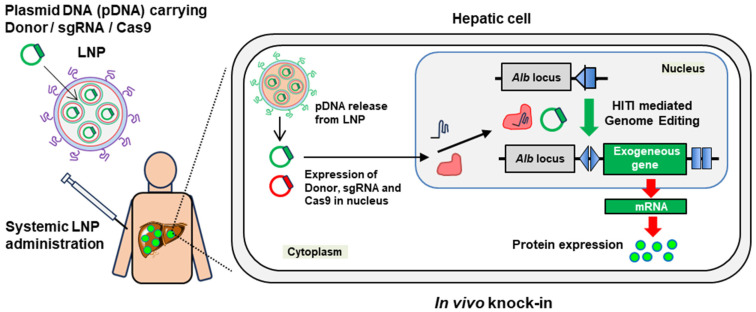
Schematic illustrating liver-targeted in vivo genome editing using LNPs in this study. Plasmid DNAs (pDNAs) carrying genome editing components (donor DNA, Cas9 and sgRNA) are delivered to the liver via LNPs. In hepatic cells, the pDNAs are released from the LNPs and transported into the nucleus. Once inside the nucleus, Cas9 and sgRNA are expressed from the pDNA, forming a Cas9/sgRNA complex that cleaves the target sequence at the *Alb* locus. As a result, the exogenous gene from the donor pDNA is inserted through a knock-in. This knock-in at the *Alb* is expected to result in high protein expression.

In this study, we advance the field by demonstrating the use of plasmid DNA (pDNA)-loaded LNPs for large DNA knock-ins in vivo (Figure 1). The liver was chosen as our target organ due to its large size, providing a substantial reservoir for therapeutic protein production, and its natural affinity for LNPs, as observed in siRNA and mRNA delivery studies [23]. A particularly promising strategy for stable long-term gene expression is targeting the albumin-coding *Alb* gene locus in hepatocytes, which is known for its robust liver-specific expression [24]. This locus offers a powerful platform for sustained therapeutic interventions [24,25].

We first compared the knock-in efficacy of HDR and HITI in hepatic cells in vitro, confirming that HITI outperforms HDR at the *Alb* locus. Next, we optimized LNP formulations for pDNA delivery and in vivo knock-in, fine-tuning parameters such as the ionizable lipid composition, formulation, and administration route. Our in vivo experiments involved intravenous administration of LNP-loaded genome-editing components into mice, resulting in successful HITI-mediated gene knock-in within the liver. Notably, the redosable nature of our system allows for a cumulative knock-in effect with repeated administration. This study marks the first successful demonstration of in vivo knock-in of large DNA using pDNA-based LNPs.

## 2. Materials and Methods

### 2.1. Plasmid Construction

To construct the SpCas9 expression pDNA (referred as “Cas9”), the Cas9 DNA fragment was amplified from hCas9 (Addgene #41815, Watertown, MA, USA) and inserted into a CMV promoter-containing pCMV backbone vector using the In-Fusion HD cloning kit (Takara Bio, Kusatsu, Japan), resulting in a final pDNA size of 7859 bp. For the NanoLuc expression pDNA (“NLuc”), the CMV promoter from the Cas9 pDNA was amplified and used to replace the PGK promoter in the pNL1.1.PGK[Nluc_PGK] Vector (Promega, Madison, WI, USA) using the In-Fusion HD cloning kit. The GFP expression pDNA for the in vivo study (“EGFP-NLS”) was constructed by inserting synthesized double-stranded DNAs encoding NLS and GFP (Genewiz, South Plainfield, NJ, USA) into a CAG promoter-containing pCAG backbone pDNA, also using the In-Fusion HD cloning kit. The 20 bp genomic sgRNA target sequence with a 3 bp PAM (underlined) of sgAlb14, targeting exon 14 of the mouse *Alb* locus, is as follows: GTTGTGATGTGTTTAGGCTAAGG [26]. Notably, efficient on-target integration and no significant off-target integration were observed with this sgRNA in the context of NHEJ-mediated in vivo knock-in, as demonstrated in the previous study [26]. To construct the sgAlb14 expression pDNA (referred as “sgAlbEx14”), the sgAlb14 sequence was inserted into the AflII (NEB, Ipswich, MA, USA) site of the sgRNA_Cloning Vector (Addgene, #41824) using the In-Fusion HD cloning kit. For the homology-directed repair (HDR), donor pDNA (referred to as “HDR-Donor”) 1.3 kb (5′) and 1.4 kb (3′) homology arms flanking the sgAlb14 target site were amplified from the mouse genome. To generate the T2A peptide-fused GFP sequence, PCR was performed using Q5 High-fidelity Polymerase (NEB) with EGFP-NLS as a template. The resulting product was inserted into a subcloning backbone pDNA using the In-Fusion HD cloning kit. The homology-independent target integration (HITI) donor pDNA (referred to as “HITI-Donor”) was constructed as previously described [10], containing the T2A-GFP sequence flanked by two sgAlb14 target sequences, generated via PCR using Q5 High-fidelity Polymerase. These fragments were subcloned into the subcloning backbone pDNA. To construct the fusion vector (referred to as “HITI-sgRNA-Donor”), which combines the HITI donor DNA and the sgRNA expression cassette, the T2A-GFP sequence flanked by two sgAlb14 target sequences and the sgRNA expression cassette (amplified from the sgAlbEx14 pDNA) were inserted into the pAAV-based backbone pDNA (Addgene, #87116) using the In-Fusion HD cloning kit. The sizes of the HDR-Donor, HITI-Donor, and HITI-sgRNA-Donor pDNAs are 699 bp, 4336 bp, and 4146 bp, respectively. All relevant sequences are listed in Appendix A Table A1, and constructs were verified by Sanger sequencing.

### 2.2. Cell Lines

Hepa1-6 (mouse hepatocarcinoma cell line) was purchased from KAC and cultured in DMEM (Wako Fujifilm, Richmond, VA, USA) containing 10% FBS (Biowest, Nuaille, France) and 1% penicillin-streptomycin solution (Gibco, Billings, MT, USA).

### 2.3. Animals

Six-week-old female Balb/c mice (Oriental Yeast) were used for in vivo transfection and knock-in experiments. The mice were housed in an approved facility with free access to food and water, individually caged in ventilation-controlled rooms under a 12-h light/dark cycle at room temperature. All animal procedures were conducted in accordance with the guidelines of the Osaka University Animal Care and Use Committee and approval of the animal study protocol by the committee (Protocol code: R2-1-2; Date of approval: 6 November 2023). Efforts were made to minimize the number of animals used and to reduce their discomfort.

### 2.4. In Vitro GFP Knock-In in Hepa1-6 Cells

Hepa1-6 cells were transfected with either the GFP donor pDNA (HDR-Donor or HITI-Donor) alone (Donor) or in combination with Cas9 and sgAlb14 expression pDNAs (Donor + Cas9). For each transfection, 1 μg of each pDNA (such as donor, Cas9, and sgAlbEx14) was used per well in 12-well plates. Transfections were performed using Lipofectamine 3000 (Thermo Fiscer, Scientific Waltham, MA, USA), where 4 μL of Lipofectamine 3000 reagent and 1 μL of P3000 reagent were mixed with 1 μg pDNA in Opti-MEM (Gibco). The transfected cells were passaged at regular intervals, and samples were collected at passages 2, 5, 8, and 11 for analysis. GFP expression (% GFP-positive cells) was assessed using a CytoFLEX S flow cytometer (Beckman Coulter, Brea, CA, USA). Cells were gated as follows. First, intact cells were selected by gating based on forward scatter area (FSC-A) and side scatter area (SSC-A) properties. Single cells were then gated using forward scatter width (FSC-Width) and FSC-A. The GFP-negative cell population was defined using dot plots from negative control (NC) cells. GFP-positive cells were detected using the GFP FITC filter, and their percentage was calculated as the number of cells outside the GFP-negative region defined by the NC cells. Genomic DNA was extracted from Hepa1-6 cells at passage 11 using the DNeasy Blood and Tissue Kit (Qiagen, Hilden, Germany) for the junction PCR assay. Additionally, Hepa1-6 cells were transfected with the fusion pDNA containing sgRNA and the GFP donor pDNA (HITI-sgRNA-Donor) alone (Donor) or in combination with the Cas9 pDNA (Donor + Cas9) and evaluated with the same procedure.

### 2.5. Junction PCR Assay

**Figure A2:**
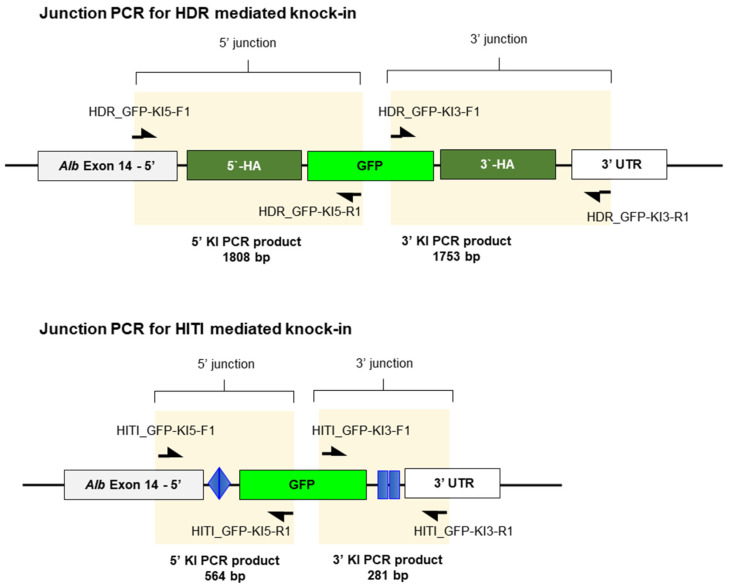
Schematic for junction PCR assay for HDR (**top**) and HITI (**bottom**)-mediated knock-in to *Alb* locus.

Genomic DNA extracted from Hepa1-6 cells or mice liver tissue was used as the template for the junction PCR assay. PCR amplification was performed using PrimeSTAR Max DNA polymerase (Takara Bio) following the manufacturer’s protocols. The primers used for the junction PCR are shown in Appendix A Figure A2 and listed in Appendix A Table A2. PCR products were separated by electrophoresis on a 1% agarose gel, and bands corresponding to the expected product sizes were identified: 564 bp for the 5′ junction and 281 bp for the 3′ junction in HITI and 1808 bp for the 5′ junction and 1753 bp for the 3′ junction in HDR-mediated genome editing.

### 2.6. LNP Preparation

All lipids used in this study were purchased from commercial suppliers: DLin-MC3-DMA (MC3), ALC-0315, and SM-102 from Cayman Chemicals, Cholesterol from Sigma-Aldrich (St. Louis, MO, USA), and DSPC and PEG2000-DMG from Avanti Polar Lipids (Alabaster, AL, USA). All LNPs used in this study were prepared by using the ethanol dilution method [27]. A representative LNP preparation procedure is as follows: 2 mL of lipid-ethanol solution is immediately added (within 3 s) through a pipette to 2 mL of pDNA solution in 25 mM citrate buffer (pH 3.5) placed in a 15 mL centrifugal tube, followed by an immediate vortex of 1 min. Then, the crude LNP suspension is incubated for 3 min at room temperature and then vortexed again for 1 min. The crude LNP suspension is diluted with Milli-Q water 10 times (adding 36 mL of Milli-Q water) and concentrated to approximately 2 mL diafiltration using Amicon Ultra Centrifugal Filters (30 kDa MWCO, Millipore, Burlington, MA, USA). This water dilution and concentration step was repeated one more time. Next, the water-replaced LNP suspension was diluted with PBS (pH 7.4) to 10 times (adding 36 mL of PBS (pH7.4)) and then concentrated back to approximately 2 mL through the diafiltration step as described above. This diafiltration step was repeated 2 times. All procedures were carried out at ambient temperature. The molar lipid ratios of the different LNP formulations, with a final pDNA concentration of 0.2 mg/mL, were as follows [17,18]:

• DLin-MC3-DMA/Cholesterol/DSPC/PEG2000-DMG = 50/38.5/10/1.5 (mol%);
• ALC-0315/Cholesterol/DSPC/PEG2000-DMG = 46.3/42.7/9.4/1.6 (mol%);
• SM-102/Cholesterol/DSPC/PEG2000-DMG = 50/38.5/10/1.5 (mol%).

The N/P ratio of LNP samples varied from 6.0 to 8.4. LNP samples were diluted 20-fold in PBS (pH 7.4), and their z-average diameter, polydispersity index, and zeta potential were measured using dynamic light scattering (Zetasizer Nano ZS, Malvern Isntrumets, London, UK). The encapsulation efficiency of pDNA in the LNPs was determined using the Picogreen assay (Thermo Fiscer) Scientific according to the manufacturer’s protocol.

### 2.7. In Vivo Transfection Study in Balb/c Mice

To evaluate the in vivo transfection efficiency of various LNP formulations (using MC3, ALC-0315, and SM-102 as ionizable lipids), luciferase (Luc) activity was measured in the major organs (liver, lung, spleen, kidney, and heart) of 6-week-old female Balb/c mice. Mice received intravenous (IV) tail vein injections of either saline (Saline) or NLuc pDNA-loaded LNPs (LNP). The pDNA dose for LNPs was 0.5 μg/g, with an injection volume of 10 μL/g. Mice were euthanized 12 h post-administration by CO_2_ inhalation, and major organs were collected. Organ lysates were prepared by homogenizing tissues in Lysis Buffer (Promega), according to the manufacturer’s protocol. Luciferase activity in the organ lysates was quantified using the Nano-Glo Luciferase Assay System (Promega) and a Synergy HTX plate reader (BioTek, Winooski, VT, USA). The ratio of luciferase activity (%) in each organ (lung, spleen, heart, and kidney), corrected by liver luciferase activity, was calculated to assess the liver specificity of transfection. For MC3-LNP, subcutaneous (SC, in the back of the neck) and intramuscular (IM, in the left quadriceps) administration routes were also tested, with an injection volume of 5 μL/g and 10 μL/g, respectively. The dosing volume for each administration route was set according to the standard volume known for the mice experiment. Specifically, for the SC route, the dosing volume is typically limited to 100 μL per mouse to avoid discomfort or pain associated with larger volumes. We adhered to this guideline to ensure the well-being of the animals. The impact of the N/P ratio was further evaluated through IV administration of EGFP-NLS pDNA-encapsulated MC3-LNP, using the same conditions as above. Hydrodynamic tail vein injection (referred to as “HD”) was employed as a positive control, with a pDNA dose of 0.5 μg/g and an injection volume of 100 μL/g. Mice were perfused with PBS 48 h post-administration. The collected livers were homogenized in PBS containing 5 mM EDTA (Dojindo, Mashiki, Japan) and 0.1% (*w*/*v*) collagenase (Wako Fujifilm) to disperse the tissues into single-cell suspensions. GFP expression was then analyzed by flow cytometry, as described in Section 2.3. The GFP-negative area was defined using the liver cell suspension from the saline-treated group of mice. Notably, hepatocytes were not specifically isolated, the analysis included a mixed cell population, encompassing both hepatocytes and non-parenchymal cells. Consequently, the percentage of GFP-positive cells reflects this heterogenous cell population from the liver sample.

### 2.8. TIDE Analysis

TIDE (Tracking of Indels by Decomposition) analysis was performed on samples collected at the first passage following in vitro transfection. These samples correspond to those used in the in vitro knock-in experiments. Analysis was conducted using the online TIDE software (https://tide.nki.nl/, accessed on 29 November 2024) [28]. Details of the PCR primers used for fragment preparation and sequencing are provided in Appendix A Table A2.

### 2.9. In Vivo Knock-In Study in Balb/c Mice

Six-week-old female Balb/c mice were utilized for the in vivo knock-in experiment to investigate sustained GFP expression through HITI-mediated knock-in delivered by MC3-LNP. The mice were divided into 6 groups and treated with tail vein injections as follows: (1) Saline (Saline), (2) GFP donor pDNA-loaded LNP alone (Donor), (3) GFP donor pDNA-loaded LNP combined with Cas9 pDNA-loaded LNP (Donor + Cas9), (4) GFP donor pDNA and Cas9 pDNA co-encapsulated in the same LNP (Donor/Cas9), (5) GFP donor pDNA- and Cas9 pDNA-loaded LNP at the double the dose used in group 3 (Donor + Cas9_2x dose), and 6) two separate injections of group 3’s formulation on day 0 and day 7 (Donor + Cas9_2x shot). The dose for both the donor pDNA and the Cas9 pDNA was 0.5 μg/g for groups 1 through 4 and 1 μg/g total for groups 5 and 6, with an injection volume of 10 μL/g for all groups. At 14 days post-treatment, mice were perfused with PBS, and the collected organs (liver, lung, spleen, kidney, and heart) were homogenized in PBS containing 5 mM EDTA and 0.1% (*w*/*v*) collagenase to disperse the tissues into single-cell suspensions. The 14-day timeline was chosen based on prior in vivo knock-in experiments conducted in our laboratory using GFP as a reporter [9]. GFP expression was then analyzed by flow cytometry. DNA and RNA were extracted from the collected organs using the AllPrep DNA/RNA Mini Kit, following the manufacturer’s protocol, and further analyzed by junction PCR and RT-qPCR assays.

### 2.10. Digital PCR

**Figure A3:**
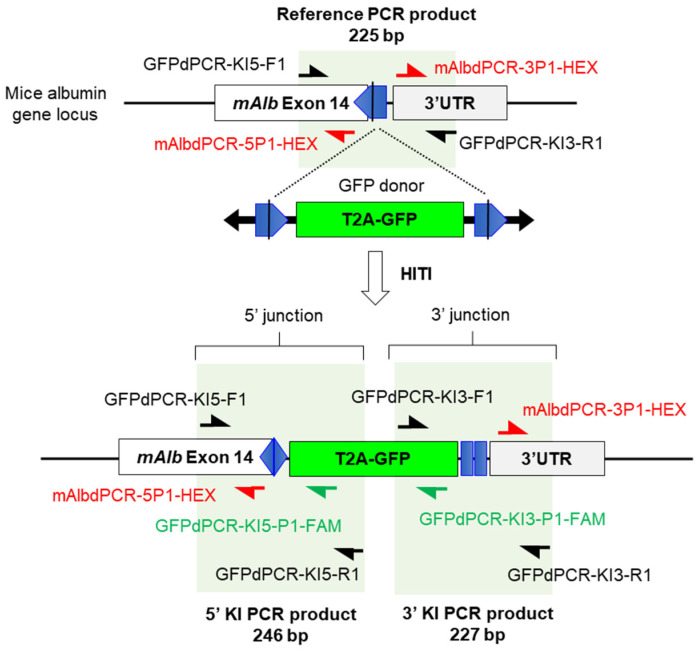
Schematic for digital PCR assay for HITI-mediated knock-in to *Alb* locus.

The QuantStudio Absolute Q Digital PCR System (Thermo Fiscer Scientific) was employed for digital PCR analysis. Genomic DNA was digested using MluCI (NEB) and used as the template for the assay. The primers and probes utilized for digital PCR are described in Appendix A Figure A3 and Table A2. The template, primers, and probes were combined with an Absolute Q DNA Digital PCR Master Mix (5×) (Applied Biosystems, Foster City, CA, USA) following the manufacturer’s protocols. The knock-in efficiency was determined using the equation

Knock-in (%) = (Copy number of target)/(Copy number of reference + Copy number of target) × 100.

### 2.11. RT-qPCR

The primers used for RT-qPCR are listed in Appendix A Table A2. Reverse transcription of RNA to cDNA was performed using the SuperScript IV First-Strand Synthesis System (Invitrogen, Waltham, MA, USA). cDNA samples were mixed with primers and SsoAdvanced Universal SYBR Green Supermix (BioRad, Hercules, CA, USA), and RT-qPCR was conducted on the CFX384 Touch Real-Time PCR System (BioRad). Data were analyzed using the ΔΔCt method, with fold changes in RNA expression levels for GFP and Cas9 calculated by normalizing the Ct values to those of the internal control (*Gapdh*) and the saline group. The fold RNA expression compared to saline was calculated as follows:

Fold RNA expression compared to Saline = 2^[{(Ct of target) − (Ct of *Gapdh*)} − {(Ct of saline sample) − (t of *Gapdh*)}]

### 2.12. Short-Term Safety Evaluation of LNP Administration

For the mice treated in the in vivo knock-in study, plasma concentrations of hepatic markers and body weight were assessed before and after administration. Commercially available assay kits were used for AST and ALT (Sigma-Aldrich) and Albumin (Fuji Rebis, Tokyo, Japan), following the manufacturer’s protocols. The absorbance of each sample was measured using a Synergy HTX plate reader.

### 2.13. Histological Analysis of Liver Slice from Knock-In Mice

Livers from treated animals were collected following perfusion with PBS and subsequently fixed in 10% formaldehyde solution. Frozen liver sections were prepared using a 2800 FRIGOCUT microtome (Reichert Jung, Cambridgeshire, UK) and immuno-stained for GFP and albumin, along with nuclear staining using DAPI (Invitrogen). For GFP immunostaining, a chicken anti-GFP IgY antibody (Aves Labs, Davis, CA, USA) was used as the primary antibody after dilution (1:200), followed by Alexa Fluor 488 goat anti-chicken IgY (Invitrogen) as the secondary antibody with dilution (1:500). For albumin immunostaining, a mouse anti-albumin antibody (Takara Bio)- was used as the primary antibody (2 μg/mL), with Alexa Fluor 555 goat anti-mouse IgG (Invitrogen) as the secondary antibody after dilution (1:500). Confocal images of the immunostaining sections were captured using a LSM800 confocal microscope (Carl Zeiss, Oberkochen, Germany).

### 2.14. Statistical Analysis

Statistical analyses were performed using Prism 10 (GraphPad, La Jolla, CA, USA). One-way ANOVA and Student’s *t*-tests were conducted to evaluate differences between groups. Bar plots were also generated using the same software.

## 3. Results and Discussions

### 3.1. Comparison of HDR- and HITI-Mediated Knock-In Efficiency in Hepatic Cells

**Figure 2:**
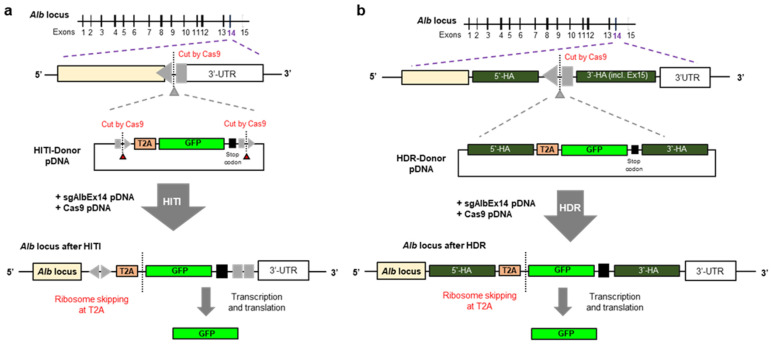
Schematic for (**a**) HITI and (**b**) HDR-mediated knock-in to the *Alb* locus.

To compare the knock-in efficiencies of HDR and HITI in hepatic cells, we designed GFP donor pDNAs for each method, specifically targeting the mouse *Alb* locus (Figure 2).

**Figure 3:**
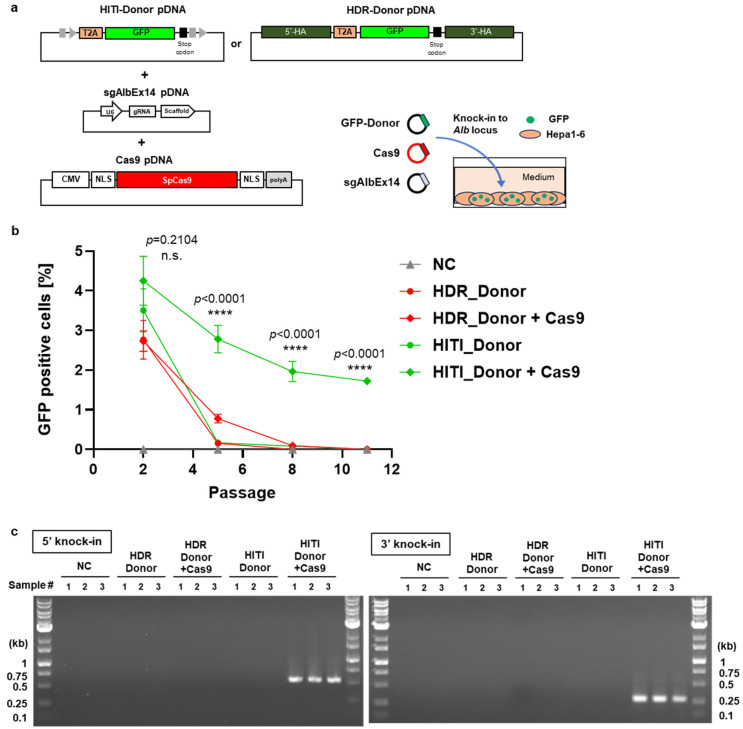
Comparison of HDR- and HITI-mediated GFP knock-in at the *Alb* locus in vitro. (**a**) Schematic representation of the in vitro transfection experiment targeting the *Alb* locus in Hepa1-6 cells. (**b**) Long-term GFP expression across multiple cell passages following HDR- and HITI-mediated knock-in. Experimental groups include the untreated control (NC), GFP donor pDNA alone (Donor), and GFP donor pDNA with Cas9 pDNA (Donor + Cas9). Data are shown as mean ± SD (*n* = 3 biological replicates). Statistically significant differences are marked by asterisks (**** *p* < 0.0001), while “n.s.” (not significant) indicates no significant difference between HITI_Donor + Cas9 and other groups except for NC, as analyzed using one-way ANOVA. (**c**) Junction PCR assay confirming successful knock-in at passage 11 via HITI, with the target band sizes of 564 bp for the 5′ junction and 281 bp for the 3′ junction. Original figures can be found in Supplementary Materials.

**Figure A4:**
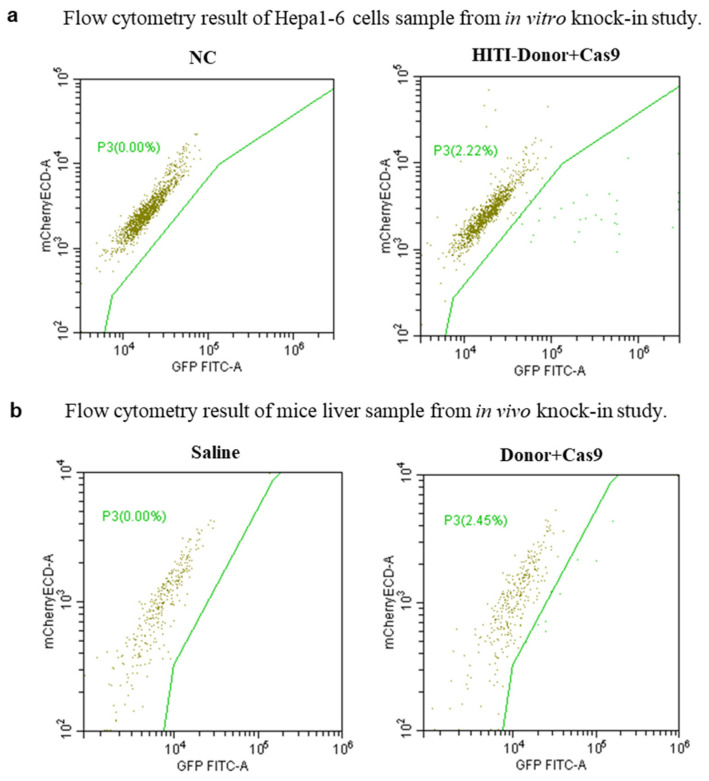
Representative dot plots from flow cytometry analysis. (**a**) In vitro knock-in study using Hepa1-6 cells (related to Figure 3b). (**b**) In vivo knock-in study in Balb/c mice (related to Figure 9a).

**Figure A5:**
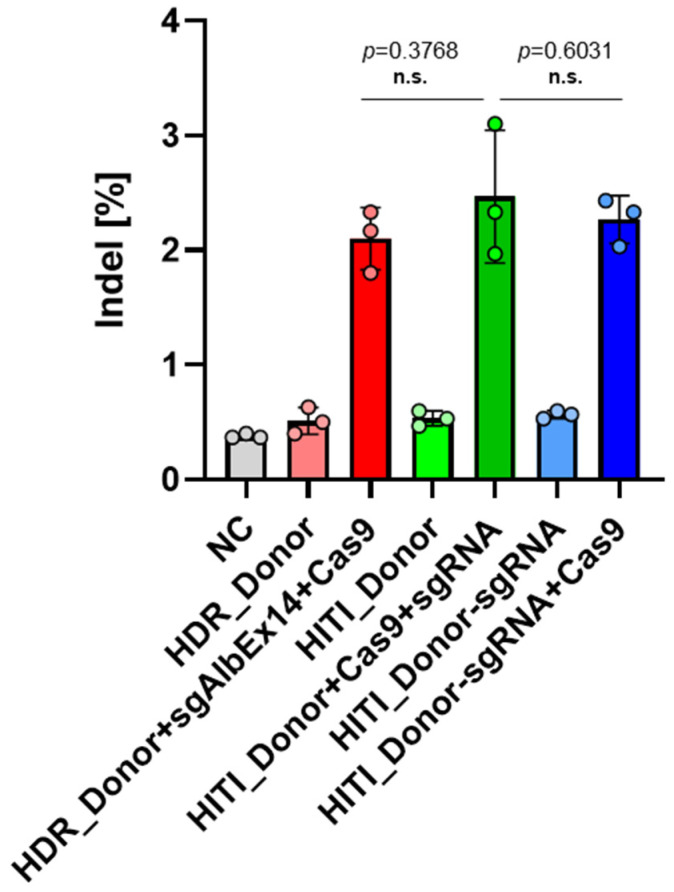
TIDE analysis results for samples collected two days after transfection in Hepa1-6 cells. Data are presented as mean ± SD (*n* = 3 biological replicates). n.s. indicates no significant difference (*p* > 0.05) among treatment groups, analyzed by one-way ANOVA.

These donor DNAs, either alone or co-transfected with Cas9- and sgRNA-expression pDNAs, were introduced into the mouse hepatocarcinoma cell line, Hepa1-6. GFP expression was monitored across multiple cell passages using flow cytometry (Figure 3a,b, and Appendix A Figure A4a and Figure A5). TIDE analysis revealed comparable indel efficiencies across groups transfected with no donor, HDR donor, or HITI donor, indicating similar Cas9/sgAlb14 cleavage activities under these conditions. In the early passages, GFP expression was detectable in the Donor-only groups (both HDR and HITI) as well as in the HDR Donor + Cas9 group. However, by passage 8, GFP expression became undetectable in these groups. In stark contrast, the HITI Donor + Cas9 group exhibited sustained GFP expression beyond passage 11, indicating successful integration and long-term gene expression.

The transient GFP signal observed in the Donor-only groups likely arose from the leaky expression of the promoter-less donor DNA, as previously reported [29]. Although the HDR_Donor and HITI_Donor differ in the presence or absence of homology arms, both donors share the same promoter-less backbone vector. Once the donor vector enters the nucleus but is not incorporated into the chromosome, unspecific transcription of the donor vector can occur, which is not regulated by a promoter, leading to the observed leaky GFP expression. Importantly, after passage 8, persistent GFP expression was only seen in the HITI Donor + Cas9 group, confirming that GFP expression at this stage was a reliable indicator of successful knock-in, as no signal was detected in the Donor-only groups during later passages.

To further confirm targeted genomic integration, junction PCR assays were performed on genomic DNA isolated from cells at passage 11. As expected, the PCR result revealed the presence of both the 5′ and 3′ junction band, in the HITI Donor + Cas9 group, confirming successful knock-in (Figure 3c). These findings demonstrate that HITI is a more efficient knock-in method compared to HDR, and it was therefore selected for subsequent in vivo liver knock-in studies in mice. Although Hepa1-6 cells are continuously dividing, and thus HDR-mediated knock-in was expected in the HDR_Donor + Cas9 group, we did not observe any GFP expression in passage 11. We attribute this result to the inherently lower HDR activity in this particular cell line. It is well known that HDR efficiency can vary significantly across different cell lines, depending on factors such as cell type, cell cycle phase, DNA repair protein expression, and the method used for delivering the HDR components [30,31,32,33].

### 3.2. Enhanced GFP Knock-In Efficiency Using the HITI-sgRNA-Donor in Hepa1-6 Cells

**Figure 4:**
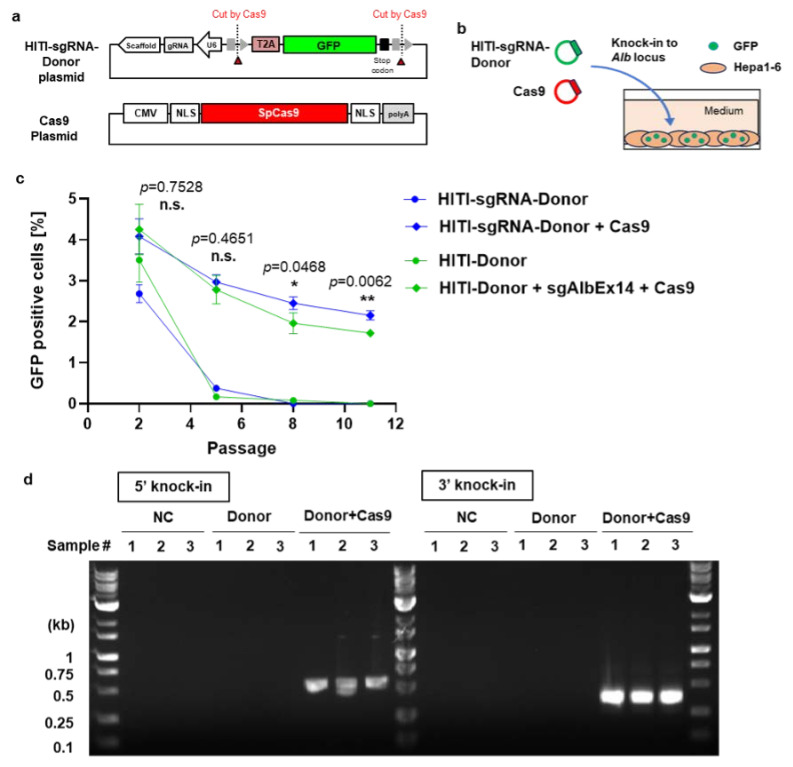
Enhanced knock-in efficacy of the HITI-sgRNA-Donor pDNA in Hepa1-6 cells. (**a**) Schematic representation of the HITI-sgRNA-Donor pDNA used as the T2A-GFP knock-in donor and the Cas9 pDNA. (**b**) Image of the in vitro transfection experiment design. (**c**) GFP expression in Hepa1-6 cells across multiple passages following HITI-mediated knock-in with either HITI-Donor or HITI-sgRNA-Donor pDNAs. Groups include untreated control (NC), GFP donor pDNA alone (Donor), and GFP donor pDNA with Cas9 pDNA (Donor + Cas9). Data are shown as mean ± SD (*n* = 3 biological replicates). Asterisks (* *p <* 0.05, ** *p* < 0.01) indicate statistically significant differences, while n.s. refers to not significantly different (*p* > 0.05) in comparison between HITI-sgRNA-Donor + Cas9 and HITI-Donor + sgAlbEx14 + Cas9, analyzed by Student’s *t*-test. (**d**) Junction PCR assay of genomic DNA from Hepa1-6 cells at passage 11 post-HITI-sgRNA-Donor transfection, confirming HITI-mediated knock-in with expected band sizes of 564 bp for the 5′ junction and 281 bp for the 3′ junction. Original figures can be found in Supplementary Materials.

To optimize pDNA delivery for in vivo knock-in, we designed a GFP donor pDNA containing an integrated sgRNA expression cassette (HITI-sgRNA-Donor) and evaluated its knock-in efficacy in Hepa1-6 cells (Figure 4a,b). In the Donor + Cas9 group, cells were co-transfected with two plasmids (HITI-sgRNA-Donor and a Cas9 pDNAs) and compared with those co-transfected with three plasmids (HITI-Donor, sgAlbEx14, and Cas9 pDNAs). Supporting data from TIDE analysis further demonstrated that the indel efficiency was comparable between 2-plasmid and 3-plasmid transfection groups, indicating similar Cas9 cleavage efficiency across conditions (Appendix A Figure A5).

While the percentage of GFP-positive cells was initially similar between the HITI-Donor and HITI-sgRNA-Donor groups, the HITI-sgRNA-Donor exhibited a significantly higher percentage of GFP-positive cells in the Donor + Cas9 group by passages 8 and 11 (Figure 4c). Junction PCR analysis at passage 11 further confirmed successful knock-in at the *Alb* locus, exclusively in the Donor + Cas9 group (Figure 4d). These findings highlight the superior efficiency of the HITI-sgRNA-Donor, leading to its selection for subsequent in vivo knock-in studies.

### 3.3. Selection of LNPs for In Vivo Transfection Studies in Mice

To advance the clinical potential of LNP-mediated genome editing, we tested three ionizable lipids: DLin-MC3-DMA (MC3), ALC-0315, and SM-102. These lipids have been integral to successful therapeutics, with MC3 used in transthyretin amyloidosis therapeutics and ALC-0315 and SM-102 in mRNA-based COVID-19 vaccines [17,34]. Despite their prominence, no direct comparison existed regarding their efficacy in liver transfection for pDNA. To identify the most effective LNPs for liver-targeted pDNA delivery, we evaluated various LNP formulations incorporating each of these ionizable lipids. The physicochemical properties of the LNPs, encapsulating either NanoLuc (Nluc) or GFP-NLS expression pDNAs, were thoroughly assessed (Table 1). All formulations displayed optimal liver-accumulation properties, including an average particle size of approximately 100 nm, weakly anionic surface charges, and high pDNA encapsulation efficiency [23].

**Figure 5:**
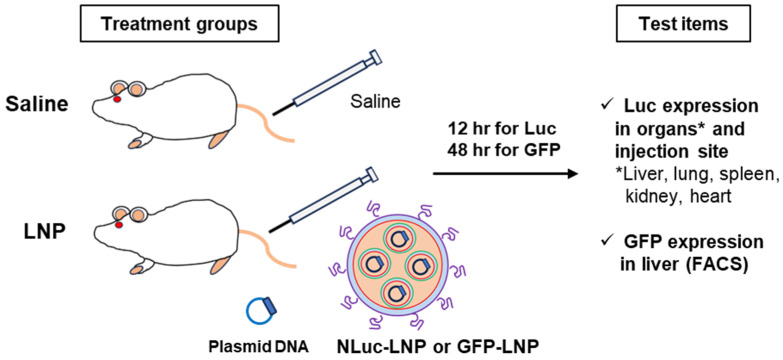
Schematic representation of the in vivo transfection study in Balb/c mice administered with NLuc and GFP expressing pDNA-loaded LNPs.

The generated NLuc expression pDNA-loaded LNPs (MC3-LNP_NLuc, ALC0315-LNP_NLuc and SM102-LNP_NLuc) were administered intravenously (IV) to Balb/c mice, and luciferase activity was subsequently measured in various organs (Figure 5).

**Figure 6:**
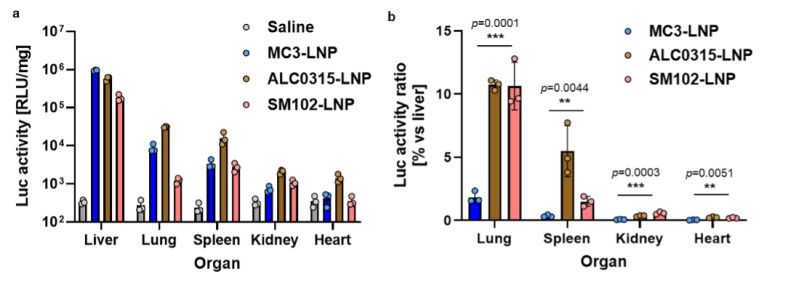
Comparative in vivo transfection efficiency of different LNP formulations. (**a**) Luciferase (Luc) activity in major organs of Balb/c mice following intravenous administration of NLuc pDNA-loaded LNPs. (**b**) Relative Luc activity in organs normalized to liver activity after intravenous delivery of various LNP formulations. Data are presented as mean ± SD (*n* = 3 biological replicates). Asterisks (** *p* < 0.01 and *** *p* < 0.001) denote statistically significant for MC3-LNP with other groups, determined by one-way ANOVA.

Among the formulations, MC3-LNP exhibited the highest transfection efficacy in the liver, outperforming both ALC0315- and SM102-LNPs (Figure 6a). Furthermore, MC3-LNP demonstrated the highest liver transfection, with minimal transfection observed in non-liver tissues compared to the other formulations (Figure 6b). These results suggest that MC3-LNP has superior liver-accumulation properties for pDNA delivery, making it a strong candidate for further in vivo studies.

**Figure 7:**
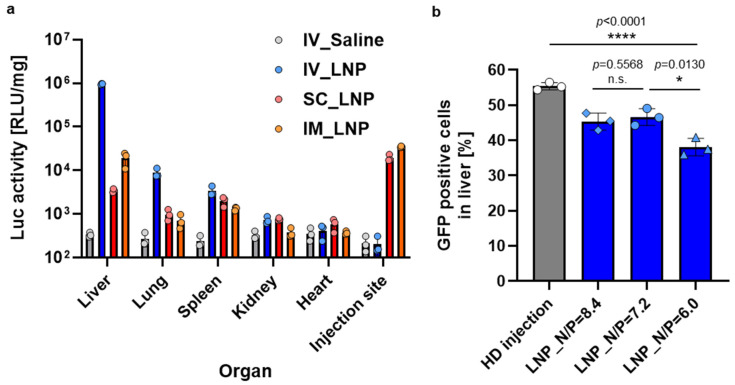
In vivo transfection efficiency of MC3-LNP. (**a**) Luc activity in various organs of Balb/c mice following intravenous (IV), subcutaneous (SC), and intramuscular (IM) administration of NLuc pDNA-loaded MC3-LNP. (**b**) GFP expression in the liver of Balb/c mice after hydrodynamic (HD) injection of a GFP expressing pDNA (EGFP-NLS) and EGFP-NLS-loaded MC3-LNP at different N/P ratios. Data are presented as mean ± SD (*n* = 3 biological replicates). Asterisks (* *p* < 0.05, **** *p* < 0.0001) denote statistically significant differences, while n.s. indicates no significant difference between groups, determined by one-way ANOVA.

We next assessed the impact of different administration routes on the tissue distribution of MC3-LNP_NLuc transfection, comparing subcutaneous (SC) and intramuscular (IM) methods, both commonly used in clinical settings, with IV administration (Figure 7a). Luciferase expression in the liver was two orders of magnitude lower for SC and IM routes compared to IV. Notably, SC and IM injections showed high luciferase expression at injection sites, indicating that the majority of LNPs stayed localized with minimal systemic distribution. These findings confirm that IV administration is the most effective route for in vivo liver transfection with MC3-LNP.

Subsequently, we optimized the MC3-LNP formulation by adjusting the N/P ratio, which represents the molar ratio of cationic charges from the amine moieties (N) in the ionizable lipid to anionic charges from the phosphate moieties (P) in the DNA. The N/P ratio is recognized as a critical material attribute for mRNA-loaded LNPs, influencing key physicochemical properties such as encapsulation capacity, particle size, and transfection efficiency [35]. Therefore, we investigated the impact of varying N/P ratios on pDNA-loaded LNPs. The physicochemical properties of the LNPs prepared with different N/P ratios were comparable across the tested formulations (Table 1). To assess the effect of the N/P ratio on transfection efficiency, we evaluated GFP expression following IV administration of EGFP-NLS pDNA-loaded LNPs at various N/P ratios using flow cytometry (Figure 7b). Hydrodynamic (HD) injection, renowned for its exceptional liver transfection efficacy but impractical for human applications due to the large injection volume, was employed as a positive control [36]. Notably, GFP expression in the liver achieved with LNPs was comparable to that observed with HD injection. Among the tested N/P ratios, both 7.2 and 8.4 demonstrated similar efficacy, significantly outperforming the 6.0 ratio. Based on these findings, we selected the N/P ratio of 7.2 for further in vivo knock-in studies, as it was anticipated to result in lower liver toxicity compared to the 8.4 ratio due to reduced lipid content.

### 3.4. In Vivo Knock-In Study in Mice

To assess the efficacy of HITI-mediated knock-in in vivo, we used the HITI-sgRNA-Donor as both the sgRNA expression vector and GFP donor. The HITI-sgRNA-Donor and Cas9 expression plasmids were encapsulated in MC3-LNP at an N/P ratio of 7.2, with two loading strategies: separately loaded or co-loaded (Table 2). All tested formulations showed optimal liver-accumulating properties [23], with no significant differences between LNPs loaded with NLuc or EGFP-NLS pDNAs, demonstrating the versatility of these LNPs for encapsulating various plasmids (Table 1 and Table 2). Moreover, the co-encapsulation of HITI-sgRNA-Donor and Cas9 pDNAs, as well as the simple mixing of separate LNPs loaded with each pDNA, did not affect the physicochemical properties of the LNPs, confirming their suitability for in vivo knock-in experiments.

**Figure 8:**
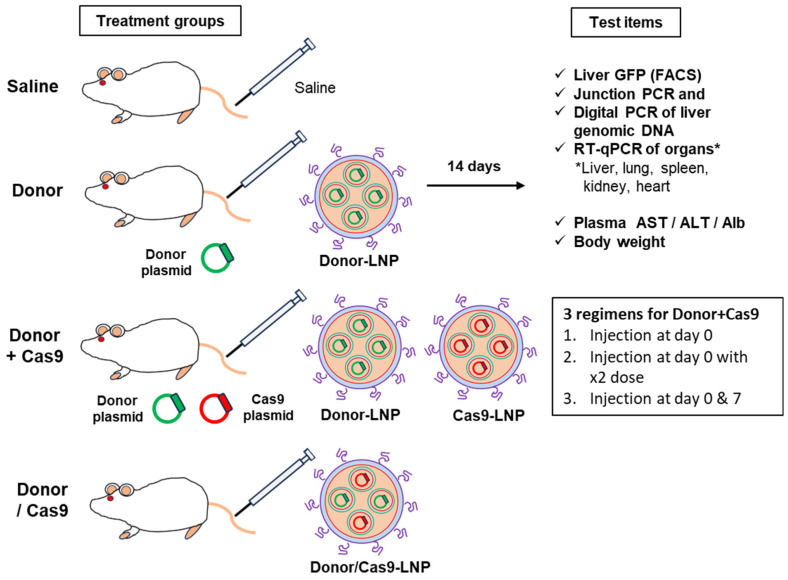
Schematic for the in vivo knock-in study with Balb/c mice administered by genome editing component-loaded LNPs.

**Figure 9:**
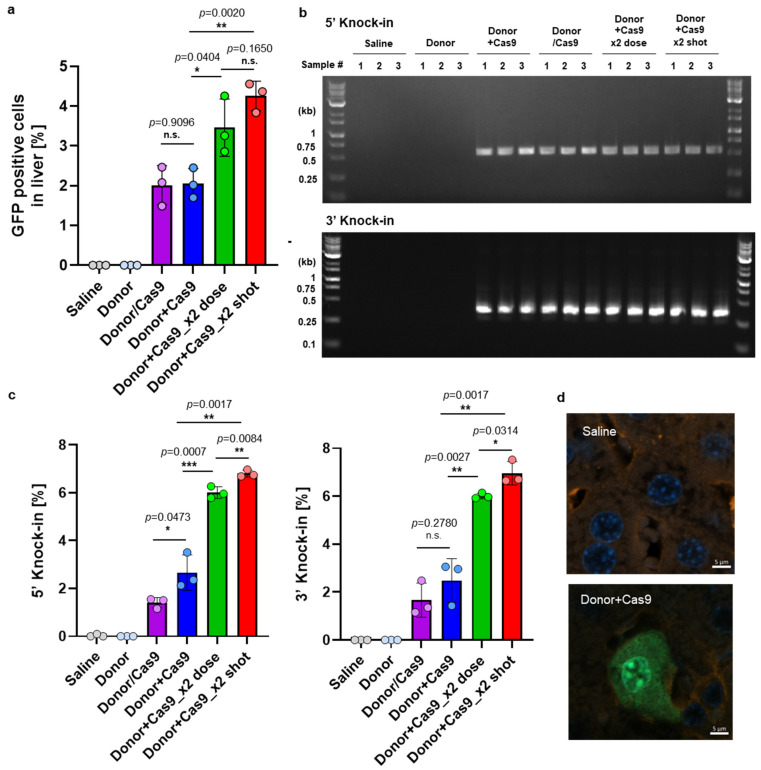
In vivo GFP knock-in study in Balb/c mice. (**a**) GFP expression in the livers of Balb/c mice following the in vivo knock-in experiment. Data are shown as mean ± SD (*n* = 3 biological replicates). (**b**) Junction PCR assay results from liver genomic DNA of treated mice, collected 14 days after the initial administration. The expected band sizes for the 5′ and 3′ knock-in junctions are 564 bp and 281 bp, respectively. (**c**) Digital PCR assay result: 5′ knock-in (**left**) and 3′ knock-in. (**d**) Confocal image of liver slices from Saline (**top**) and Donor + Cas9 (**bottom**)-treated mice. Asterisks (* *p* < 0.05, ** *p* < 0.01, *** *p* < 0.001) denote a statistically significant difference, while n.s. indicates no significant differences between groups, determined by one-way ANOVA. Original figures can be found in Supplementary Materials.

Mice were intravenously administered genome-editing components encapsulated in MC3-LNP, and their livers were harvested 14 days post-administration for analysis of GFP expression by flow cytometer (Figure 8). No GFP expression was observed in the Saline and Donor-only groups, while the Donor + Cas9 group exhibited approximately 2% GFP-positive cells (Figure 9a and Appendix A Figure A4b). Notably, co-encapsulation of the GFP donor/sgRNA and Cas9 pDNAs (Donor/Cas9) yielded GFP levels comparable to those achieved with separately loaded LNPs containing the GFP donor/sgRNA and Cas9 pDNAs (Donor + Cas9), indicating the flexibility of delivery methods for HITI-mediated genome editing components.

Doubling the pDNA dose in the Donor + Cas9 group (Donor + Cas9_2x dose) significantly increased GFP expression, from 2.1% to 3.5% (*p* < 0.01). Additionally, employing a repeated dosing strategy, where Donor + Cas9 was administrated on day 0 and day 7 (Donor + Cas9_2x shot), further elevated GFP expression in the liver to 4.3%, nearly doubling that of the single-dose Donor + Cas9 group (2.1%). Interestingly, no significant difference was found between the repeated dosing (4.3%) and the single administration with the doubled pDNA dose (3.5%) (*p* > 0.05), suggesting that flexible dosing regimens could be tailored to therapeutic needs. Junction PCR confirmed the presence of target-size bands in the genomic DNA from the livers of mice treated with both the Donor and Cas9 co-treated groups (Figure 9b). While repeated LNP dosing has been reported for gene knockout in local tissues, such as muscle [37], our study represents the first demonstration of successful in vivo knock-in using repeated systemic LNP administration.

To quantify the knock-in efficacy at the genomic level, digital PCR analysis was conducted. No knock-in was observed in the Saline and Donor-only groups, while the Do-nor + Cas9 group exhibited knock-in efficiency of 2.5–2.7% at both 5′ and 3′ junction, and 6.0–7.0% for double dose and twice administered animal group at both 5′ and 3′ junction (Figure 9c). The trend in knock-in efficacy closely mirrored the GFP expression data, suggesting that increasing the dose or frequency of administration can enhance in vivo knock-in efficiency. To note, approximately 3% HITI-mediated gene knock-in in mouse liver was achieved by using AAVs [9], a figure comparable to the efficiency observed with the LNP-based approach in this study. This highlights that AAV and LNP methods can achieve similar knock-in efficiencies in the liver. Additionally, histology data of liver slices from both non-treated and knock-in mice were obtained (Figure 9d). Since albumin is known as a representative marker for hepatocytes [38], both albumin and GFP were detected and observed to investigate where the knock-in occurred. The confocal image results suggested that knock-in occurs mainly in hepatocytes rather than other non-parenchymal cells.

**Figure 10:**
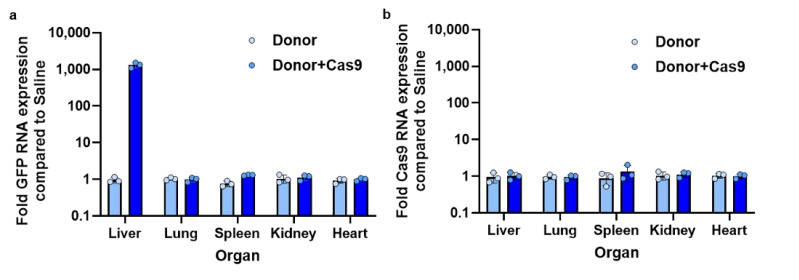
RT-qPCR analysis of GFP and Cas9 RNA expression levels from the in vivo knock-in study. (**a**) Fold GFP RNA expression compared to saline-treated mice in various organs of mice treated with Donor and Donor + Cas9, 14 days post-administration. (**b**) Fold Cas9 RNA expression compared to saline-treated mice in various organs of mice treated with Donor and Donor + Cas9, 14 days post-administration. Data are present as mean ± SD (*n* = 3 biological replicates).

To further assess liver-specific GFP expression via knock-in, we performed RT-qPCR analysis on RNA extracted from various organs. The results revealed GFP RNA expression exclusively in the liver of Donor + Cas9-treated mice at 14 days post-administration (Figure 10a). While sustained Cas9 expression has been associated with an increased risk of off-target mutations [39,40], no significant increase in Cas9 RNA levels was detected at 14 days post-administration (Figure 10b). These findings suggest that Cas9 expression was transient, enabling effective liver-specific GFP knock-in while likely minimizing the risk of off-target effects due to prolonged Cas9 activity.

**Figure 11:**
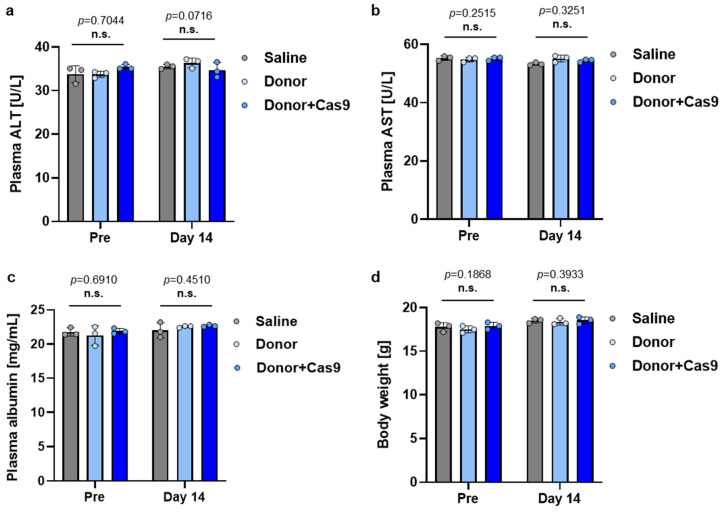
Short-term safety assessment of HITI-mediated knock-in via LNP administration. (**a**) Plasma ALT levels measured in treated mice. (**b**) Plasma ALT levels measured in treated mice. (**c**) Plasma albumin concentration measured in treated mice. (**d**) Body weight of treated mice was measured before and 14 days after administration. Data are presented as mean ± SD (*n* = 3 biological replicates). n.s. indicates no significant difference (*p* > 0.05) among treatment groups, analyzed by one-way ANOVA.

Finally, the safety of LNP administration was evaluated by measuring plasma ALT and AST levels, both of which are key markers for hepatotoxicity, along with plasma albumin concentration and body weight, before and 14 days after LNP administration. Plasma albumin levels were assessed to ensure that knock-in at the *Alb* locus did not disrupt normal albumin production. No significant differences were observed between the treatment groups (Saline, Donor, and Donor + Cas9), either before and or 14 days post-administration (Figure 11). These results indicate that LNP-mediated HITI knock-in does not induce acute hepatotoxicity and supports the short-term safety of this approach for in vivo genome editing.

Taken together, these findings demonstrated that HITI-mediated knock-in using MC3-LNP offers an efficient, flexible, and safe approach for genome editing in the liver. While GFP was used as a model gene in this study, the method can be easily adapted for applying other therapeutic genes, as LNPs are not constrained by the size limitations of AAV vectors, which are restricted to sequences smaller than 4.7 kb [41]. Future research may extend this knock-in technology to organs beyond the liver. LNPs are well known for their ability to modulate biodistribution, allowing the targeting of organs such as the lungs or liver through adjustments in lipid composition [42]. This flexibility presents promising opportunities for genome editing across a broader range of tissues and diseases.

## 4. Conclusions

In this study, we developed knock-in pDNAs targeting the *Alb* locus and demonstrated that HITI significantly outperforms HDR as a more efficient method for knock-in in hepatic cells. Among the LNP formulations tested, MC3-LNP emerged as the most effective vehicle for intravenous liver transfection. We achieved GFP knock-in in mice liver through co-administration of GFP donor/sgRNA pDNA-loaded MC3-LNPs alongside Cas9 pDNA-loaded MC3-LNPs. Notably, no knock-in was observed when only the GFP donor/sgRNA pDNA-loaded LNPs were administered, underscoring the critical role of Cas9 in this process. Additionally, repeated dosing of the GFP/sgRNA and Cas9 pDNA-loaded LNPs resulted in a two-fold increase in the liver GFP-positive cells, suggesting a cumulative knock-in effect. The short-term safety of LNP-mediated knock-in was also confirmed, with no signs of increased hepatotoxicity, reduced plasma albumin levels, or changes in body weight in treated mice. Although LNP transfection was observed in non-liver tissue, such as the lung and spleen (Figure 6a and Figure 7a), we believe the concern regarding off-target activity is minimal, based on the limited Cas9 RNA detected in the liver (where the highest transfection was observed) and the absence of significant safety concerns at 2 weeks post-administration (Figure 10b and Figure 11). However, off-target effects and their impact on safety and efficacy will require more extensive analysis in future clinical translations of LNP-based genome editing.

Given that the potential for LNPs to deliver large pDNAs remains largely underexplored, previous liver knock-ins have primarily relied on viral vectors like AAV or hybrid approaches combining LNPs for Cas9 and sgRNA delivery with AAV for donor DNA delivery [6,22,23]. This study represents the first successful demonstration of in vivo knock-in of large DNA using LNPs alone, without the need for viral vectors. The versatility of pDNA-loaded LNPs also opens doors for other knock-in approaches, such as PITCh method, which leverages the microhomology-mediated end joining (MMEJ) repair pathway, or Prime editing, which bypasses the need for a donor DNA template [43,44,45]. Furthermore, LNPs could be adapted for genome editing systems that require high-molecular-weight proteins, such as the CRISPR-Type I system (Cas3), which functions with a multi-protein complex called Cascade [46]. These findings lay the groundwork for future applications of LNP-mediated knock-in of large genetic fragments, particularly for therapeutic protein production within the body.

## Figures and Tables

**Table 1:** Physicochemical properties of LNPs used in the in vivo transfection study.

LNP Sample	Loaded pDNA	N/P Ratio	Z-Average Diameter [nm]	PDI *	Zeta Potential [mV]	Encapsulation Efficiency [%]
MC3-LNP_NLuc	NLuc	8.4	142	0.089	−4.35	95.5
ALC0315-LNP_NLuc	NLuc	8.4	137	0.159	−4.47	98.8
SM102-LNP_NLuc	NLuc	8.4	140	0.187	−3.49	96.2
MC3-LNP_EGFP_8.4	EGFP-NLS	8.4	136	0.137	−3.22	95.1
MC3-LNP_EGFP_7.2	EGFP-NLS	7.2	141	0.120	−5.15	92.6
MC3-LNP_EGFP_6.0	EGFP-NLS	6.0	138	0.120	−4.31	93.5

* PDI: Polydispersity index.

**Table 2:** Physicochemical properties of LNPs used in the in vivo knock-in study.

LNP Sample	Loaded pDNA	Z-Average Diameter [nm]	PDI *	Zeta Potential [mV]	Encapsulation Efficiency [%]
MC3-LNP_Donor-GFP	HITI-sgRNA-Donor	142	0.089	−4.35	95.5
MC3-LNP_Cas9	Cas9	137	0.159	−4.47	98.8
MC3-LNP_Donor-GFP/Cas9 **	HITI-sgRNA-Donor Cas9	141	0.120	−5.15	92.6
MC3-LNP_Donor-GFP + MC3-LNP_Cas9 ***	HITI-sgRNA-Donor Cas9	138	0.120	−4.31	93.5

* PDI: Polydispersity index. ** Co-encapsulation of HITI-sgRNA-Donor and Cas9 pDNAs into MC3-LNP. *** Mixture (1:1 ratio by pDNA amount) of HITI-sgRNA-Donor pDNA encapsulated in MC3-LNP and Cas9 pDNA encapsulated in MC3-LNP.

## Data Availability

The original contributions presented in the study are included in the article/Supplementary Material; further inquiries can be directed to the corresponding author.

## Supplementary Materials

The following supporting information can be downloaded at: https://www.mdpi.com/article/10.3390/biom14121558/s1, File S1: Original gel electrophoresis pictures.

## Appendix A

**Table A1:** Primers used for the construction of pDNAs.

Primer	Objective	Sequence (5′ to 3′)
sgAlb14gRNA-F	Construction of sgRNA expression pDNA	TGGCTTTATATATCTTGTGGAAAGGACGAA ACACCGTTGTGATGTGTTTAGGCTA
sgAlb14gRNA-R	Construction of sgRNA expression pDNA	GCCTTATTTTAACTTGCTATTTCTAGCTCTA AAACTAGCCTAAACACATCACAAC
T2AGFP-HDR-F	Construction of HDR GFP donor pDNA	GACGATGACGATAAGTTCGAAGGAAGGGGC TCTTTGCTTACTTGTG
T2A-GFP-HDR-R	Construction of HDR GFP donor pDNA	TGGTTCTAGACTCGATCACTT GTACAGCTCGTCCATGCCG
T2AGFP-HITI-F	Construction of HITI GFP donor pDNA	CCTTAGAGGGCGTTTAAACGTTGTGATGTG TTTAGGCTAAGGTTGAAGGAAGGGGCTCTT TGCTTACTTGTG
T2AGFP-HITI-R	Construction of HITI GFP donor pDNA	CGCGTGCGGCCGCAGCCTTAGCCTAAACA CATCACAAC TAAGATGGTGGAGGGGCGC

**Table A2:** Primers used for PCR-based assays.

Primer	Objective	Sequence (5′ to 3′)
HITI_GFP-KI5-F1 =GFPdPCR-KI5-F1	Junction PCR_5′ junction, Digital PCR_5′ junction	AAGTGCAAATCCTAACAGTCC
HITI_GFP-KI5-R =HDR_GFP-KI5-R1	Junction PCR_5′ junction	TGCCGTCCTCCTTGAAGTCGATG
HITI_GFP-KI3-F1 =HDR_GFP-KI3-F1	Junction PCR_3′ junction	CTGCTGCCCGACAACCACTACCTG
HITI_GFP-KI3-R1	Junction PCR_3′ junction	GCTTGTCTGTATGGCTCT
HDR_GFP-KI5-F1 =mAlbTIDE-PCR-F1	Junction PCR_5′ junction, TIDE_PCR fragment	GGGTGTGACTTTTGAGAATGGAGTAA GAAACAGCTGGAA
HDR_GFP-KI3-R1 =mAlbTIDE-PCR-R1	Junction PCR_3′ junction, TIDE_PCR fragment	ACCTTTAAAGCCATTCCTATCTCTTTT GTCCCCAACTCA
mAlbTIDE-Seq-F1	TIDE_Sequence primer	GAATCATTTCACATTCCCTCCC
GFPdPCR-KI5-R1	Digital PCR_5′ junction	CGCTGAACTTGTGGCCGTTT
GFPdPCR-KI3-F1	Digital PCR_3′ junction	AACGAGAAGCGCGATCAC
GFPdPCR-KI3-R1	Digital PCR_3′ junction	GCTTGTCTGTATGGCTCT
GFPdPCR-KI5-P1-FAM	Digital PCR_GFP_5′ probe	CAGCTCCTCGCCCTTGCTCAC
GFPdPCR-KI3-P1-FAM	Digital PCR_GFP_3′ probe	TCACTTGTACAGCTCGTCCAT
mAlbdPCR-5P1-HEX	Digital PCR_Control_5′ probe	TCCCAAGTATAGTTACCTGAGAAGGTT
mAlbdPCR-3P1-HEX	Digital PCR_Control_3′ probe	CTTTGTTTTCAGGGTCCAAACCTT
GFP-qPCR-F1	RT-qPCR_GFP	CTGCTGCCCGACAACCACTACCTG
GFP-qPCR-R1	RT-qPCR_GFP	ACACCAACAGAAAAGATGAGTCCTGA
Cas9-qPCR-F1	RT-qPCR_Cas9	GCAGCCAGATCCTGAAAGAACACCC
Cas9-qPCR-R1	RT-qPCR_Cas9	AGTTCCTGGTCCACGTACATATCCC
mGAPDH-F	RT-qPCR_GAPDH	CATGGCCTTCCGTGTTCCTA
mGAPDH-R	RT-qPCR_GAPDH	CCTGCTTCACCACCTTCTTGAT
