# Supplementary figures and images for: Lipid Nanoparticles Enable Efficient In Vivo DNA Knock-In via HITI-Mediated Genome Editing
Source: Biomolecules. 2024 Dec 6;14(12):1558. doi: 10.3390/biom14121558 (PMC11673532; doi:10.3390/biom14121558)

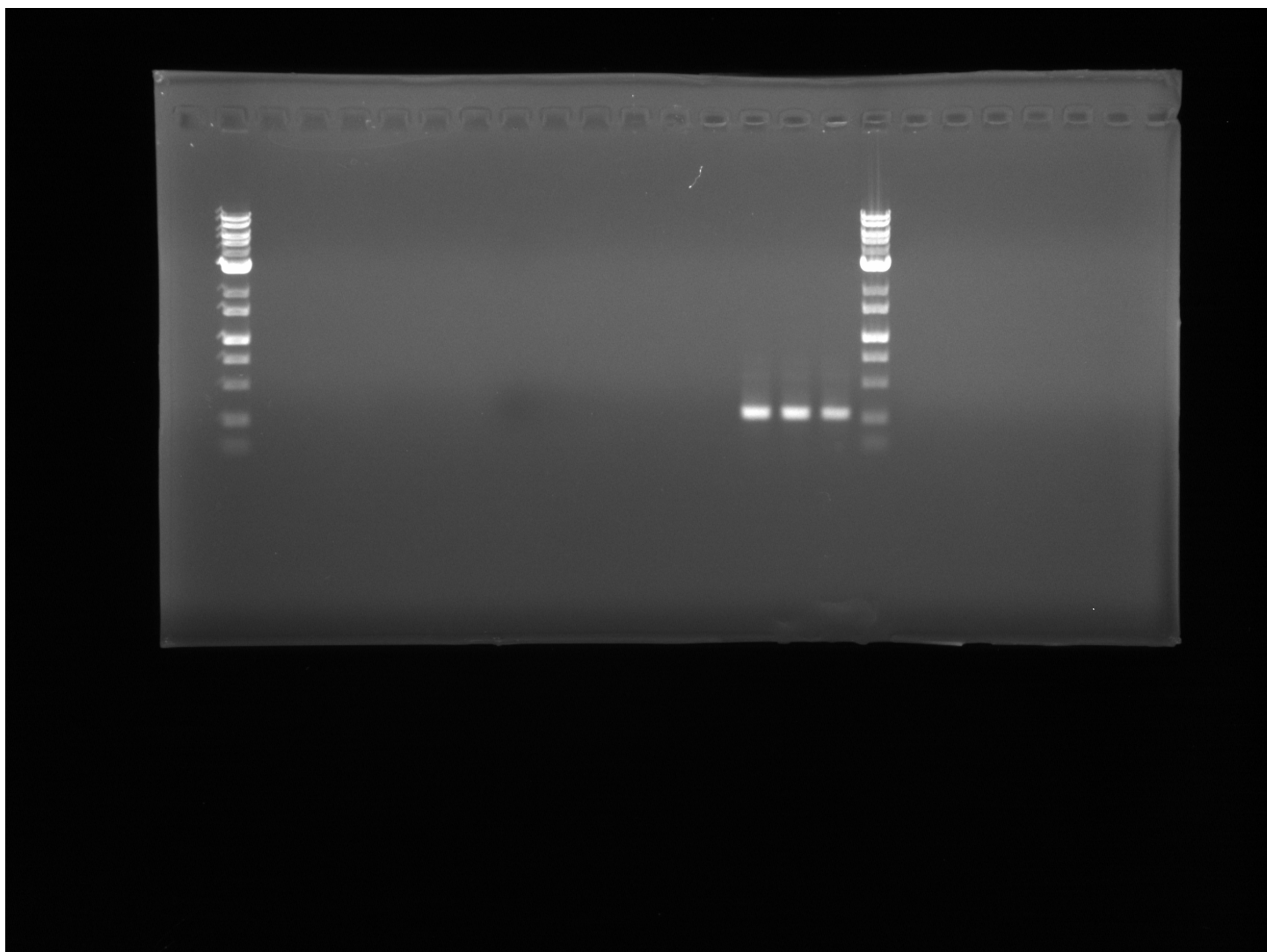

Supplement: Supplementary file 1 [file biomolecules-14-01558-s001.zip › Fig3c_Electrophoresis_3-knock-in.pdf]

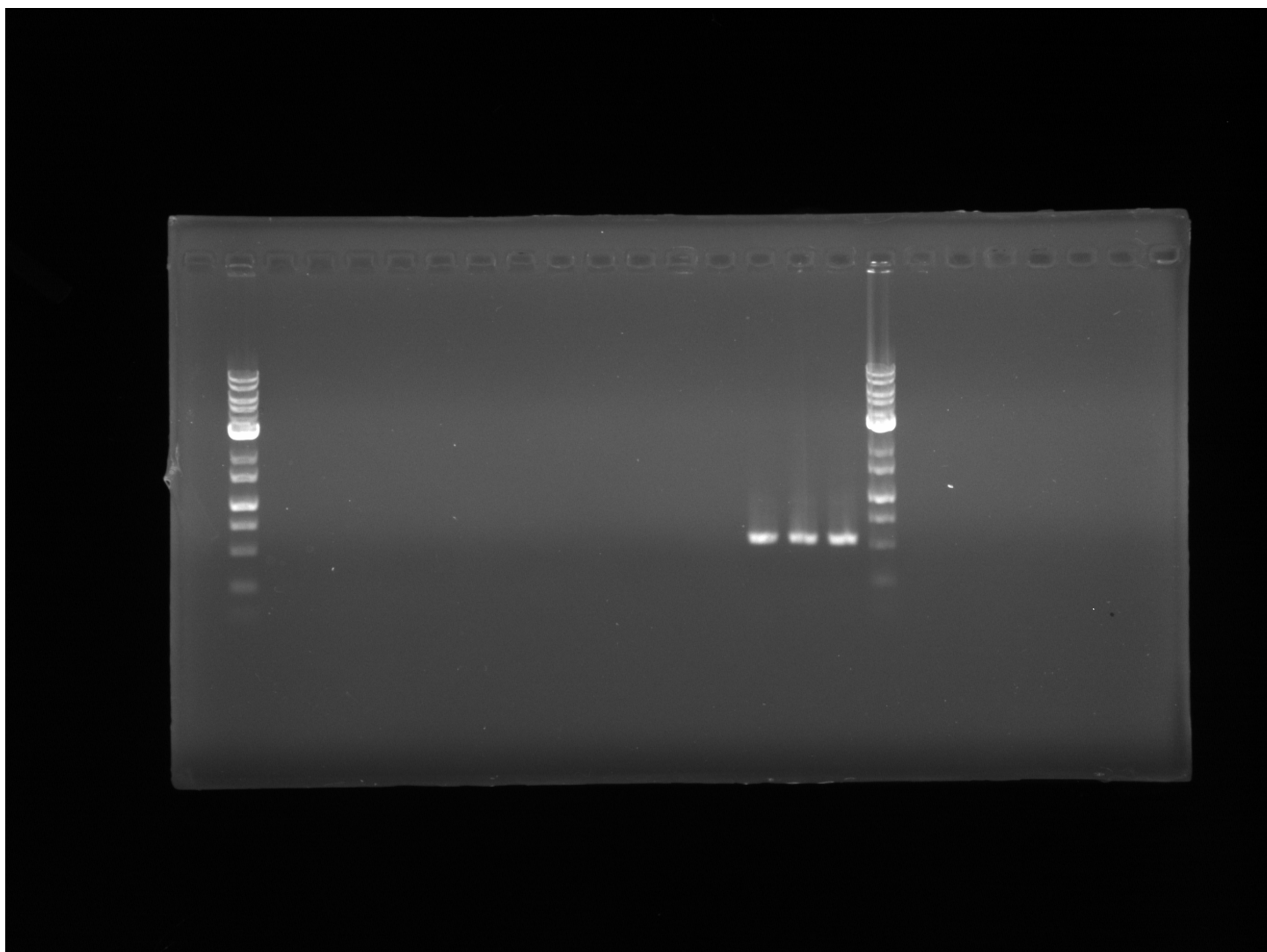

Supplement: Supplementary file 1 [file biomolecules-14-01558-s001.zip › Fig3c_Electrophoresis_5-knock-in.pdf]

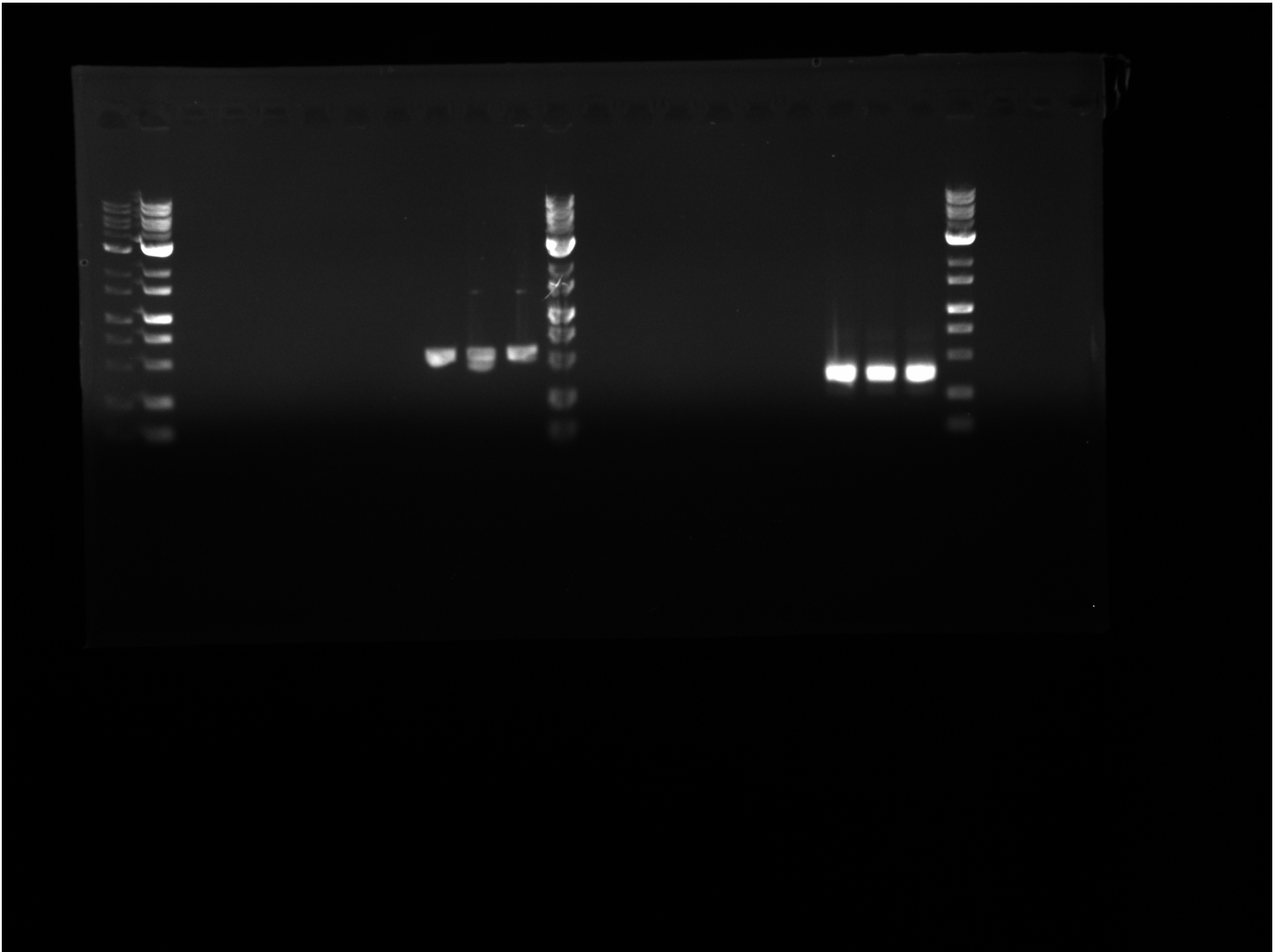

Supplement: Supplementary file 1 [file biomolecules-14-01558-s001.zip › Fig4d_Electrophoresis.pdf]

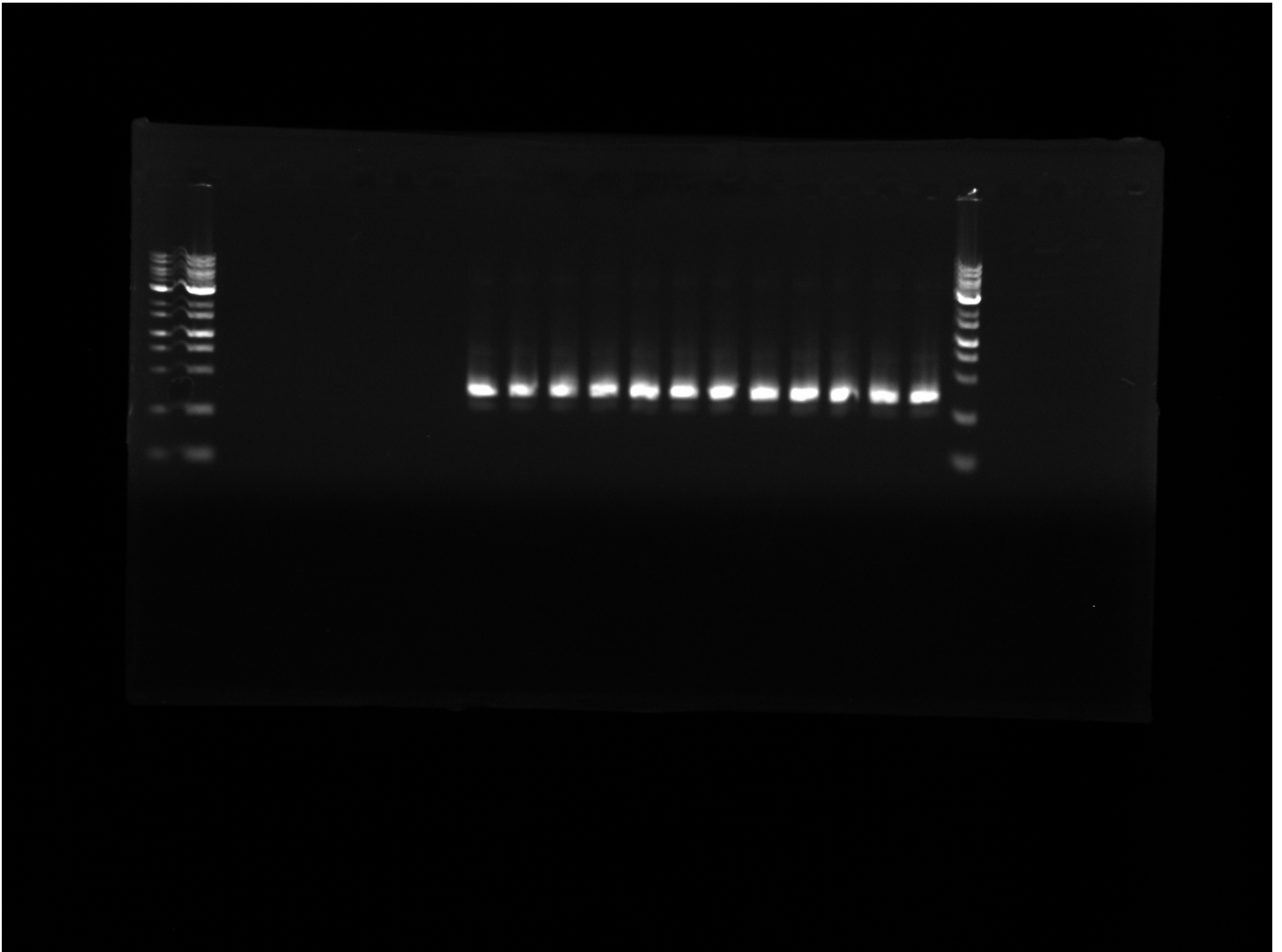

Supplement: Supplementary file 1 [file biomolecules-14-01558-s001.zip › Fig9b_Electrophoresis_3-knock-in.pdf]

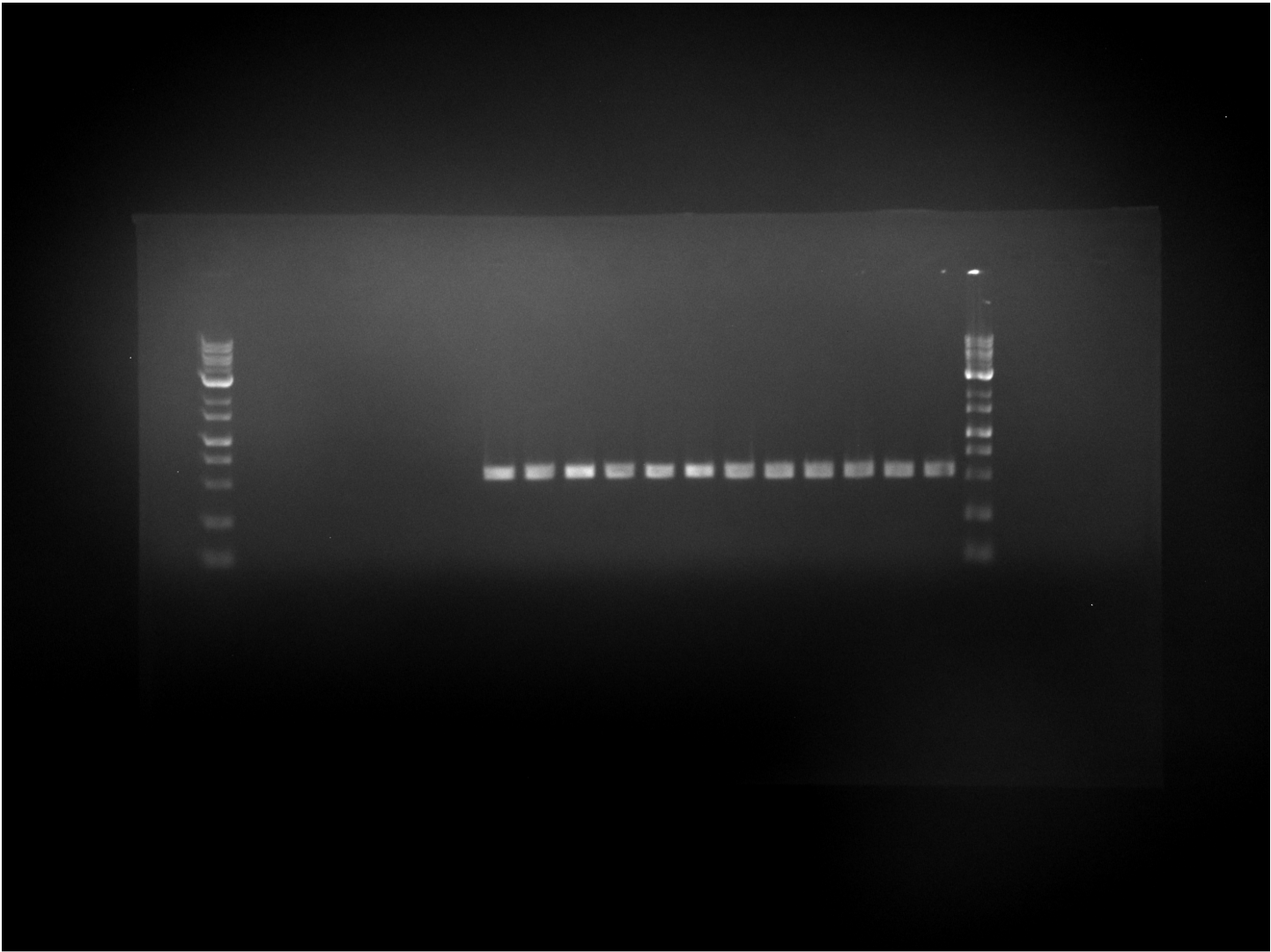

Supplement: Supplementary file 1 [file biomolecules-14-01558-s001.zip › Fig9b_Electrophoresis_5-knock-in.pdf]
